# Estradiol and Micronized Progesterone: A Narrative Review About Their Use as Hormone Replacement Therapy

**DOI:** 10.3390/jcm14207328

**Published:** 2025-10-16

**Authors:** Martina Foschi, Giulia Groccia, Maria Laura Rusce, Celeste Medaglia, Claudia Aio, Alessandra Sponzilli, Veronica Setti, Christian Battipaglia, Alessandro D. Genazzani

**Affiliations:** 1 Center for Endocrinological Gynecology, Department of Obstetrics and Gynecology, University of Modena and Reggio Emilia, 41121 Modena, Italy; martifoschi62@gmail.com (M.F.); giulia.groccia@gmail.com (G.G.); mlaura.rusce@gmail.com (M.L.R.); celemeda@gmail.com (C.M.); claudia.aio86@gmail.com (C.A.); alessandra.sponzilli@gmail.com (A.S.); settivero@gmail.com (V.S.); christianbattipaglia@gmail.com (C.B.); 2 Clinical and Experimental Medicine PhD Programme, Department of Biomedical, Metabolic and Neural Sciences, University of Modena and Reggio Emilia, 41121 Modena, Italy

**Keywords:** menopause, menopausal symptoms, hormone replacement therapy (HRT), bioidentical hormones, estradiol, micronized progesterone

## Abstract

Hormone replacement therapy (HRT) currently represents the first-line treatment to manage and reduce menopausal symptoms. Standard regimens generally combine 17β-estradiol (E2) or conjugated equine estrogens (CEEs) with micronized progesterone (P4) or synthetic progestins. While synthetic progestins ensure endometrial protection against estrogen-induced stimulation of the endometrium, their impact on metabolic, cardiovascular, skeletal, and cognitive systems is heterogeneous and not always beneficial. In contrast, progesterone, as a micronized preparation (P4), allows for more physiological effects because it is chemically identical to endogenous progesterone. This narrative review provides an updated overview of the clinical benefits of HRT regimens based on E2/P4, with a focus on their impact on endometrial thickness, venous thromboembolism (VTE), cardiovascular diseases (CVDs), breast cancer risk, cognitive effects, bone protection, and quality of life (QoL).

## 1. Introduction

Life expectancy greatly improved during the last century, thus enabling a longer lifespan for women and making them live for one-third of their lifespan in menopause. Although menopause is generally a natural process, there is now a growing need for therapies that promote healthy aging, characterized by a lower risk of disease, preserved cognitive and physical function, and active social involvement.

**Figure 1:**
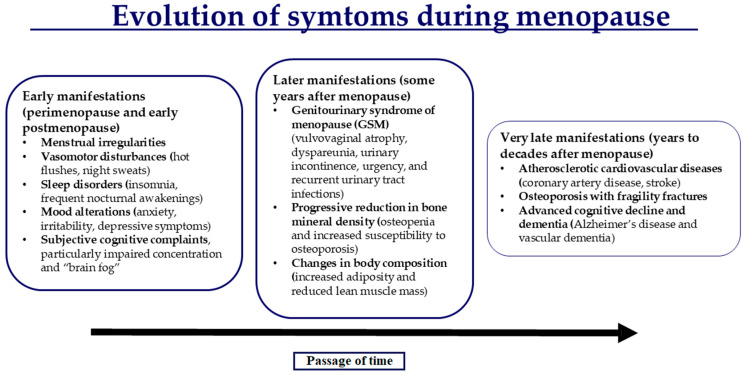
Changes during the menopausal transition are triggered by a hypoestrogenic condition that affects the central nervous system, mainly the hypothalamic area, and other organs, triggering early to late occurrence of symptoms.

Menopause is a programmed biological event that typically starts around 50 years of age, and it is defined as the absence of menstruation for at least 12 consecutive months. It occurs with a range of early and mid-term symptoms, as well as late-onset biological conditions linked to prolonged hormone deficiency. Early symptoms include vasomotor disturbances, such as hot flushes and night sweats, sleep disorders (insomnia; frequent nocturnal awakenings), mood alterations (anxiety, irritability, and depressive symptoms), and subjective cognitive complaints, with particularly impaired concentration and “brain fog”. Later complications include genitourinary syndrome of menopause (GSM), characterized by vulvovaginal atrophy, dyspareunia, urinary incontinence, urgency, and recurrent urinary tract infections, as well as progressive reduction in bone mineral density, leading to osteopenia and increased susceptibility to osteoporosis, and changes in body composition. Very late manifestations, occurring from years to decades after menopause, can include atherosclerotic cardiovascular diseases such as coronary artery disease and stroke; osteoporosis with fragility fractures; and advanced cognitive decline, including Alzheimer’s disease and vascular dementia (Figure 1). These effects are not only attributable to hypoestrogenism secondary to reduced ovarian function but also to alterations in central neurotransmitter systems, whose synthesis is regulated by estrogenic activity on both neurons and glial cells [1,2].

HRT currently represents the most effective treatment for managing and reducing menopausal symptoms. HRT with only estrogen is prescribed to women without a uterus, whereas combined estrogen–progestin therapy is used for women with an intact uterus to protect the endometrium from hyperplasia and cancer, as well as to control bleeding [2,3].

E2 and CEE were the first estrogens clinically used for HRT. Estetrol (E4), a natural estrogen produced by the fetal liver during pregnancy, is expected to be introduced soon as a potential option for menopausal treatment. Unlike E2 or ethinylestradiol (EE), E4 is a native estrogen with tissue-selective activity, acting as an agonist or antagonist depending on the tissue [4].

**Figure 2:**
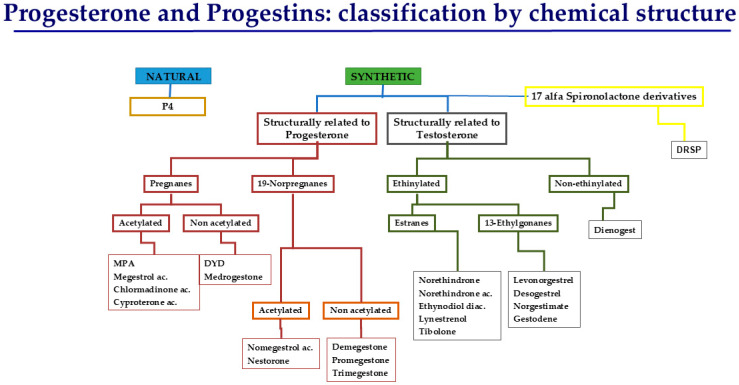
Classification of progesterone and progestins by chemical structure.

**Figure 3:**
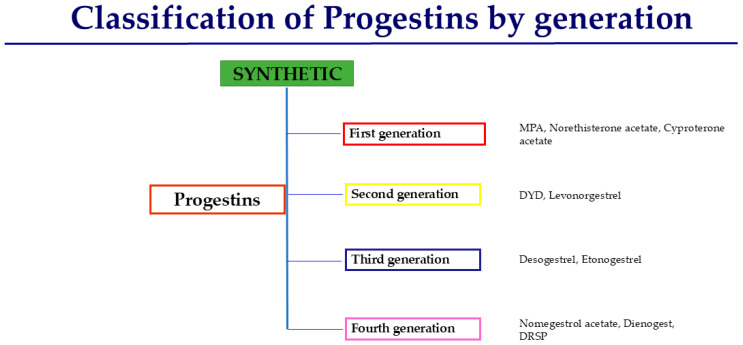
Classification of progestins used in HRT by generation.

Progestogens are essential in combined HRT to protect the endometrium against hyperplasia and cancer and to regulate bleeding [3]. Progestogens are a heterogeneous group of steroid hormones that share the ability to bind and activate the progesterone receptor, thereby eliciting biological effects comparable to those of natural progesterone. This term includes both P4 and synthetic progestins [5]. P4 is molecularly identical to the hormone physiologically secreted by the corpus luteum and the placenta. When administered in its micronized form, it selectively binds to the progesterone receptor and provides endometrial protection while exerting a relatively neutral or even beneficial influence on cardiovascular and metabolic parameters [6]. Synthetic progestins, by contrast, are structurally modified molecules developed to enhance oral bioavailability and metabolic stability. Progestins are derived from different chemical classes (Figure 2 and Figure 3), and while they effectively prevent estrogen-induced endometrial hyperplasia, many progestins also interact with androgen, glucocorticoid, or mineralocorticoid receptors, leading to variable off-target effects [7].

This leads to biological effects that are quite different from those of endogenous progesterone [7] (Table 1): DYDROGESTERONE (DYD), a progesterone derivative, has no androgenic effects and a minimal impact on metabolic parameters such as lipid profiles. It also does not significantly affect sex hormone-binding globulin (SHBG) [7]. Testosterone derivatives have good oral bioavailability and strong progestogenic effects on the endometrium. They stabilize the menstrual cycle effectively but may cause variable systemic androgenic effects [7]. Spironolactone derivatives, especially drospirenone (DRSP), have anti-mineralocorticoid and anti-hypertensive effects by blocking the action of angiotensin II and increasing sodium and water excretion. DRSP may, therefore, be preferred in women with borderline hypertension or fluid retention [7,8].

The selection of the appropriate progestin for HRT, considering its receptor profile and potential impact on other organs, enables treatment to be tailored to the patient’s specific requirements. Following the publication of the results of the Women’s Health Initiative (WHI) study, which focused on HRT involving the use of CEE and medroxyprogesterone acetate (MPA), a combination that is no longer used for menopausal treatment in Europe, concerns were raised regarding the safety of HRT [9,10]. Consequently, many women discontinued HRT, and many others remain reluctant to use it [11].

In recent years, research into bioidentical hormone use in HRT has increased, indicating that these drugs may be more effective and associated with a lower risk of breast cancer and cardiovascular disease than synthetic equivalents [12].

Bioidentical hormones are defined as compounds that are chemically identical to human endogenous steroid hormones produced by the ovaries, the adrenal gland, and the thyroid, such as 17β-estradiol, progesterone, dehydroepiandrosterone (DHEA), and levothyroxine. Bioidentical hormones used for HRT in menopause can be administered through different routes (e.g., oral, transdermal, or vaginal) and are either available as standardized pharmaceutical formulations (approved by the FDA or EMA), such as E2 or P4, or as galenic preparations, commonly referred to as “compounded bioidentical hormone therapies” [13,14]. The latter are often promoted as safer and more “natural” alternatives; however, because they are not subject to rigorous quality control, they present relevant concerns regarding purity, potency, and efficacy. For this reason, authorities, including the Endocrine Society, the North American Menopause Society, and the American College of Obstetricians and Gynecologists, discourage their use outside of clinical trials [2,13,15]. On the other hand, FDA- and EMA-approved bioidentical formulations provide standardized pharmacokinetic profiles and safety data, and their risk–benefit balance is comparable to that of other hormone therapies with synthetic progestins [2,14].

This narrative review provides an updated overview of the clinical benefits of HRT regimens based on E2/P4, with a focus on their impact on endometrial thickness, venous thromboembolism (VTE), cardiovascular diseases (CVD), breast cancer risk, cognitive effects, bone protection, and quality of life (QoL).

## 2. Research Strategy

We conducted an extensive literature search using the PubMed, Google Scholar, and Scopus databases. Our search string included all field variations of the words “menopause”, “hormone replacement therapy”, “bioidentical hormones”, “micronized progesterone”, and “estradiol”. Several abstracts were screened to identify articles relevant to our narrative review, which focus on hormone replacement therapy with bioidentical hormones, providing insights into its action in different tissues and organs. Exclusion criteria applied were languages other than English and a lack of full-text access through our institutions’ resources. From this process, a total of 103 papers were included in this study.

## 3. Endometrial Thickness, Hyperplasia, and Endometrial Cancer

As previously mentioned, unopposed estrogen stimulation of the endometrium in women with an intact uterus can lead to endometrial hyperplasia; to avoid this event, progestogens are required to protect the endometrium [16]. Several factors must be considered when using progestogens for endometrial protection in HRT, including the type, duration of administration, and dose of progestin, as well as the potency of each progestin.

Progestogen is typically administered for at least 10–14 days per month, depending on the duration of estrogen exposure [17]. The dose of progestogen needed to prevent hyperplasia depends on its potency. Some authors have concluded that 19-norpregnenolone derivatives are the most potent, followed by MPA, DYD, and P4 [18].

HRT is usually prescribed sequentially, while continuous combined therapy is offered to women who wish to avoid menstrual bleeding. Several studies have shown that continuous combined regimens provide better endometrial protection than sequential regimens [17].

The differences between P4 and progestins, as well as among different progestins, have been evaluated in various studies.

The REPLENISH trial demonstrated endometrial protection with continuous combined HRT consisting of a single daily tablet containing 1 mg of E2 with 100 mg of P4. After one year, the incidence of endometrial hyperplasia was below 1%, meeting the U.S. Food and Drug Administration (FDA)’s criteria for endometrial safety [19].

A 2018 European study reported a similar rate of increased endometrial thickness above 5 mm in women receiving E2 combined with either 100 mg of P4 or 4 mg of MPA, sustaining similar efficacy of P4 and progestins [20].

Other randomized studies, including the Postmenopausal Estrogen/Progestin Interventions (PEPI) trial [21], have shown that adding 200 mg of P4 to estrogen therapy similarly counteracts estrogen’s effects on endometrial thickness, hyperplasia, and carcinoma risk when compared with progestins such as medroxyprogesterone acetate (10 mg), DYD (10 mg), nomegestrol acetate (NOMAC) (5 mg), and chlormadinone acetate (10 mg) [16,17,18,19].

## 4. Abnormal Uterine Bleeding

Sequential HRT keeps the endometrium under control. Oral HRT is known to provide better control of bleeding than transdermal administration, with bleeding patterns generally improving over time. The amount of uterine bleeding tends to decrease after two or three treatment cycles, and the proportion of women who become amenorrheic gradually increases [13]. Bleeding is also less frequent in postmenopausal women than in perimenopausal women, and cumulative amenorrhea rates are higher in women already in menopause than in those not yet in a menopausal state [22,23].

Several studies have evaluated differences in bleeding profiles between P4 and progestins, as well as among various progestin types.

Two randomized trials showed that women receiving cyclic P4 (200 mg) combined with CEE (0.625 mg) experienced shorter bleeding days [24,25], lighter menstrual flow [23,25], and higher rates of amenorrhea [24] than those using MPA (5 mg).

Conversely, two other randomized studies found that cyclic P4 (200 mg) combined with transdermal estradiol (50 µg) was associated with more frequent irregular bleeding compared to cyclic MPA (10 mg) [26], NOMAC (5 mg) [26], DYD (10 mg) [26], and chlormadinone acetate (10 mg) [27]. Amenorrhea rates were 37.1% with P4 and 11.9% with chlormadinone acetate in one study [27], while in another study, amenorrhea rates were similar across all progestins [26].

A 2020 systematic review confirmed that oral therapy with 1 mg E2/100 mg P4 is associated with a more favorable bleeding profile than most other oral or transdermal formulations containing progestins [18].

Although the mechanisms underlying these different bleeding patterns remain unclear, it has been suggested that certain progestins induce changes in endometrial microvascular structure, favoring bleeding [28]. In vitro studies have also shown that P4 has a minor impact on the delicate balance between angiogenic and anti-angiogenic factors in endometrial glands and stroma compared to synthetic progestins [29,30].

## 5. Cardiovascular and Thrombotic Outcomes

### 5.1. VTE and Coagulation Factors

Both the route of estrogen administration and the type of estrogen and progestin used significantly influence thrombotic risk in postmenopausal women undergoing HRT.

A 2019 UK analysis of two nested case–control studies examined the association between HRT and VTE. The study included 5795 women with VTE and 21,670 controls who had been exposed to HRT within 90 days prior to the index date. Many women in both groups used oral therapy, which was associated with an increased risk of VTE compared with no HRT exposure. This increased risk was observed for both estrogen-only preparations and combined estrogen–progestogen therapies. Estradiol-based treatments were associated with a lower risk than conjugated equine estrogens, whether used alone or in combination with a progestogen [31].

Regarding the effect of progestins on VTE, three large observational studies, the Estrogen and Thromboembolism Risk Study (ESTHER), E3N, and MEVE, reported increased VTE risk with HRT containing norpregnane derivatives, but not with progesterone or pregnane derivatives [32,33,34].

Among specific combinations, conjugated equine estrogens with medroxyprogesterone acetate carried the highest risk, whereas E2 combined with DYD was associated with the lowest [35].

A 2023 retrospective cohort study confirmed a lower VTE risk in women treated with E2/P4 compared to those treated with CEE/MPA [35]. A meta-analysis revealed that while the overall risk of VTE is approximately doubled in women using oral estrogen therapy (RR 1.9), no increased risk (RR 1) was observed with transdermal estrogen administration, although there was some variability across studies [36]. This finding was further supported by the previously mentioned 2019 UK analysis, which confirmed that transdermal preparations (estradiol only or combined) were not associated with an increased risk of venous thromboembolism [31] (Table 2). It is important to note that, even when estrogen is administered via the transdermal route, the type of progestogen added can influence the impact on venous thromboembolism risk [31]. Data on the effects of transdermal estradiol combined with various oral progestins or P4 on coagulation factors are consistent with clinical observations regarding VTE risk [37,38]. A French study found that norpregnane-derived progestins were associated with reduced activated protein C (APC) sensitivity and higher plasma prothrombin levels [37], while another trial showed that progestins increase thrombin production [38]. No significant changes in these parameters were observed with P4 in either study [37,38].

### 5.2. Lipid Metabolism

HRT also significantly affects lipid metabolism. Several studies, including the Heart and Estrogen/Progestin Replacement Study (HERS) and the WHI, have shown that HRT increases triglyceride levels but substantially reduces total cholesterol and LDL cholesterol while increasing HDL cholesterol. The LDL–HDL cholesterol ratio decreases in women receiving HRT, making it anti-atherogenic. Moreover, elevated HDL levels counteract triglyceride increases, neutralizing their negative impact. Overall, HRT reduces atherosclerotic risk based on lipid profiles [39].

Differences based on the type of progestin used have been highlighted. Four randomized studies showed that P4 has no adverse lipid effects in postmenopausal women.

In the PEPI trial, women receiving cyclic CEE/P4 had HDL cholesterol levels comparable to those on CEE alone, but significantly higher levels than women receiving cyclic or continuous CEE/MPA. All regimens reduced LDL cholesterol and increased triglycerides compared to placebo [21]. Similarly, another study reported significant increases in HDL and reductions in LDL with CEE combined with either P4 (100 mg) or DYD (10 mg), while triglyceride levels increased only with DYD [40].

In a study using estradiol valerate (E2V), HDL cholesterol remained stable when combined with P4 but decreased significantly with MPA, while triglyceride levels remained unchanged in both groups [20].

In the end, a study on transdermal estradiol combined with P4 or chlormadinone acetate showed minimal effects on total cholesterol and triglycerides [27].

The route of administration of HRT significantly influences lipid metabolism, with notable differences between oral and transdermal estradiol formulations. Oral HRT undergoes extensive first-pass hepatic metabolism, leading to elevated levels of estrone and estrone sulfate in the circulation [41]. This hepatic processing can modulate liver protein synthesis, resulting in increased levels of high-density lipoprotein (HDL) cholesterol and low-density lipoprotein (LDL) cholesterol, while potentially elevating triglyceride (TG) concentrations [42,43].

Conversely, transdermal HRT bypasses the hepatic first-pass effect, delivering estradiol directly into the systemic circulation. This route maintains a more physiological estradiol-to-estrone ratio and exerts a more neutral effect on lipid profiles, particularly regarding TG levels [42,44].

Consequently, transdermal HRT may be preferable for postmenopausal women at high risk for cardiovascular disease or dyslipidemia [42].

### 5.3. Cardiovascular (CVD) and Cerebrovascular Diseases

HRT with transdermal or oral formulations combined with non-androgenic progestins has a beneficial impact on glucose–insulin metabolism, lipid profile, and blood pressure, key factors in the development of atherosclerosis. Atherosclerosis, together with a prothrombotic state, underlies the risk of heart attack and ischemic stroke. HRT appears to offer protection against these conditions, contributing to a reduction in related mortality.

A 2023 meta-analysis of all available studies showed that long-term HRT use (over 10 years) is associated with a reduction in overall mortality and in coronary heart disease mortality. When HRT starts later in life, these benefits are not observed, but no harm has been demonstrated regarding the onset of cardiovascular events [2,45]. However, this meta-analysis also revealed a slight increase in the risk of deep vein thrombosis (DVT) associated with HRT use. This risk can be mitigated by using transdermal formulations, as the thrombotic effect is mainly related to the hepatic impact of orally administered estrogens, which increase angiotensinogen levels and alter coagulation factors. These hepatic effects are not seen with transdermal HRT [45].

Considering the role of the route of administration, Canonico et al. showed how the risk of stroke onset depends mainly on the method of estrogen administration: while oral administration seems to increase stroke risk, transdermal formulation has no such effect [46].

However, regarding the role of different progestins, this case–control study also found that women using transdermal estradiol in combination with norpregnane derivatives had an increased risk of ischemic stroke. No increased risk was observed among women who used estradiol in combination with progesterone, pregnane, or nortestosterone derivatives [46].

## 6. Breast Cancer

Breast cancer risk is the main reason why many women choose not to start or discontinue HRT, partly due to extensive media coverage of the slight risk increase observed with CEE/MPA in the WHI study, negatively impacting public perception [9].

Several observational studies comparing the effects of HRT containing E2 versus CEE on the breast have yielded inconclusive and inconsistent results, with no clear difference observed between CEE and E2 users [47,48,49,50,51,52]. A retrospective analysis showed a reduced risk of breast cancer with the use of E2 or CEE alone [50], whereas a more recent study found no association between HRT containing E2 or CEE and breast cancer risk [52].

Since the 1980s, it has been widely hypothesized that the tumorigenic risk induced by estrogens is significantly amplified by progesterone due to its physiological effects on breast tissue [53]. This hypothesis was initially based on studies evaluating cell proliferation during different phases of the menstrual cycle and epidemiological observations linking breast cancer incidence to menstrual irregularities. It was later reinforced by data from HRT. However, epidemiological studies measuring serum progesterone levels in the luteal phase suggest that adequate endogenous progesterone production in premenopausal women may protect against the subsequent development of breast cancer [54]. Therefore, the absence of an increased risk observed when P4 was added to HRT, as demonstrated in the E3N study, is biologically plausible [54]. The increased risk reported in other studies is mainly related to the specific progestins used in the countries where those studies were conducted. These progestins have different actions from endogenous progesterone and may enhance the proliferative stimulus of estrogens on mammary and estrogen-sensitive tumor cells [54].

When current HRT users were compared to never-users, the data showed an increased risk of breast cancer in groups receiving P4 or DYD, as well as other progestins. Conversely, when past users of hormonal therapies were compared to never-users, an increased risk was associated with prior use of estrogen plus synthetic progestins, but not with combined estrogen and P4 or DYD therapy [54,55].

Regarding HRT and breast cancer subtype, an increased risk of lobular breast cancer was associated with CEE/DYD use and with all analyzed subtypes with E2/CEE combined with other progestins, whereas the combination of E2 and P4 showed a neutral effect on the selection of ductal or lobular breast cancer subtype [56].

Finally, two meta-analyses confirmed that the use of P4 is associated with a lower risk of breast cancer compared to other progestins [57,58]. It should be noted that this data originates from epidemiological studies.

## 7. Bone Protection

Postmenopausal osteoporosis is the most common form of primary osteoporosis. According to the National Osteoporosis Risk Assessment (NORA) study [59], 7.2% of 200,160 postmenopausal women analyzed had undiagnosed osteoporosis associated with fractures. The estrogen deficiency linked to menopause can accelerate the loss of trabecular bone mass, leading to fragility fractures predominantly involving vertebrae and distal radius [60,61]. Osteoporotic fractures cause disability in about two-thirds of affected women and increase mortality risk by 20% within the year following diagnosis [62].

Estrogens play a key role in regulating bone metabolism by inhibiting osteoclast-mediated bone resorption, promoting osteoblastic activity, and modulating growth factors, such as the receptor activator of nuclear factor kappa-B ligand (RANKL), which is crucial for osteoclast differentiation [63]. Although progestogens are less effective than estrogens at increasing bone mineral density (BMD), the literature indicates that bone protection is enhanced in women receiving combined hormonal therapy [62].

Estrogen administration, alone or in combination with progestogens via various routes, remains a valid method for osteoporosis prevention in postmenopausal women without contraindications to HRT [62].

A systematic review and meta-analysis of 28 randomized controlled trials (RCTs) showed that HRT based on either E2 or CEE reduces fracture risk compared to a placebo, with significantly greater reductions observed with E2 than with CEE [64].

Direct comparisons of HRT containing P4 versus progestins revealed no significant differences in BMD. In the PEPI trial, the use of CEE combined with P4 or MPA significantly increased spinal and hip BMD over three years compared to baseline, in contrast to the declines seen in placebo groups. Furthermore, continuous CEE plus MPA therapy was found to be more effective than cyclic MPA/P4 regimens in increasing spinal BMD [21]. Three RCTs reported BMD and bone metabolism improvements without significant differences between groups using transdermal E2 combined with continuous P4 or MPA, or oral cyclic CEE combined with P4 or DYD [65,66,67].

In summary, HRT containing E2 and P4 is effective in improving bone health and BMD, reducing the risk of fractures, with outcomes comparable to those observed with other HRT formulations.

## 8. Effects on the Central Nervous System (CNS)

Subjective cognitive decline, as a result of reduced gonadal hormone production, is one of the most reported symptoms experienced by women during menopause. It affects working memory, attention, processing speed, and verbal memory, with an estimated prevalence of between 44% and 62% in population-based studies [68,69]. Alterations in estrogenic and androgenic balance cause functional changes and limbic system-related disorders such as anxiety and insomnia, mood disturbances, migraine and headache, depressive states, asthenia, reduced libido, progressive memory loss, and eventually Alzheimer’s disease.

The incidence of Mild Cognitive Impairment (MCI) was reported at 4.5% in 6376 postmenopausal women followed for 5.4 years in the Women’s Health Initiative Memory Study (WHIMS) [70]. However, the relationship between MCI and menopausal factors remains insufficiently investigated.

Estrogens appear to exert a neuroprotective role through various mechanisms, including modulation of neurotransmitters, neurosteroid synthesis and activity [71,72], neuronal growth and synaptic plasticity [73], reduction in cellular apoptosis [74], antioxidant properties and vascular recruitment [75], and decreased β-amyloid formation [76].

The identification of biomarkers for Alzheimer’s disease (AD) in middle-aged women has strengthened the hypothesis that the decline in estrogen levels during the menopausal transition causes cognitive decline in perimenopausal women and increases their risk of dementia, which is markedly higher than that observed in men.

Recent evidence also indicates that the rise in follicle-stimulating hormone (FSH), which is a hallmark of the postmenopausal period, may contribute to the increased incidence of Alzheimer’s disease in women. FSH has been shown to act directly on hippocampal and cortical neurons, promoting amyloid-β and Tau accumulation and impairing cognition in murine models of Alzheimer’s disease. Inhibition of FSH signaling in these models prevents the development of Alzheimer’s-like phenotypes by suppressing the neuronal C/EBPβ–δ-secretase pathway. These findings not only support a causal role of elevated serum FSH levels in exacerbating Alzheimer’s disease pathology during menopause but also highlight a potential therapeutic strategy. Specifically, the use of a targeted anti-FSH antibody could represent a promising approach for future treatment of Alzheimer’s disease [77].

LeBlanc et al. [78] reviewed epidemiological data on the neuroprotective effects of HRT: symptomatic menopausal women receiving hormone therapy showed improvements in memory, vigilance, reasoning skills, and fluent speech. The same meta-analysis of observational studies on HRT and cognitive functions suggested a significant reduction in AD risk among women using hormone therapy [78].

The strongest evidence linking hormone therapy to reduced AD incidence comes from two cohort studies: the Manhattan Study of Aging and the Baltimore Longitudinal Study of Aging [79,80], both reporting a significant decrease in AD risk among estrogen-treated women.

Observational data from the Cache County Study indicate a reduced AD risk in women who used HRT for 3 to 10 years. The gender difference in AD risk disappears in women who used HRT for more than ten years [81].

Numerous observational studies and meta-analyses comparing different types of estrogen on cognitive outcomes show better results with E2 than with CEE. A meta-analysis of 36 randomized controlled trials suggested a trend (*p* = 0.1) toward slightly worse cognitive performance with CEE compared to E2 [82].

Furthermore, data on postmenopausal women with risk of dementia or Alzheimer’s disease (AD), who were treated with HRT for at least one year, showed better cognitive outcomes with E2 versus CEE [83,84,85], including significantly improved verbal memory [83,84] and higher cerebral metabolism compared to CEE users [83].

Moreover, recently, Baik et al. published a large epidemiological study focusing on HRT administration in women after 65 years of age, showing that E2 monotherapy use beyond age 65 years was associated with significant risk reductions in dementia, while estrogen and progestogen combo-therapy (both with P4 and progestins) did not have this positive impact [86]. This demonstrated that there are still unanswered questions about the role of HRT in dementia, and more studies will surely need to be performed to further investigate the link between hormone use and CNS alterations.

### Neuroendocrine Changes Under HRT

Progesterone, which is sensitized by estrogens for uptake in specific CNS areas, regulates multiple neuropsychological and neuroprotective functions in women. This action is mediated by nuclear receptors (PR-A and PR-B isoforms) and membrane receptors [87]. Both isoforms are present in the CNS, particularly in the cerebral cortex, hippocampus, amygdala, cerebellum, locus coeruleus, and glial cells. This highlights progesterone’s involvement in cognitive functions, mood regulation, and memory [88].

Progestins modulate the production, release, and metabolism of numerous neurotransmitters and their receptors. In addition, progesterone is a precursor of allopregnanolone that competes with GABA in binding GABA A receptors [88]. All these compounds interact with membrane receptors that regulate opioid, catecholaminergic, serotonergic, and GABAergic tone. This results in anxiolytic, sedative, analgesic, and anticonvulsant effects [88].

However, unlike P4, progestins differ in their impact on the CNS, partly due to their variable capacity to convert into allopregnanolone, a metabolite that binds to GABA receptors [89].

19-nortestosterone derivatives have a lower potential to increase allopregnanolone levels, as they are not fully metabolized into progesterone and subsequently allopregnanolone [90].

DRSP appears to have no effect on allopregnanolone production in either the brain or serum, indicating a neutral neurometabolic profile [91].

DYD increases allopregnanolone concentration in a dose-dependent manner in the frontal cortex, hippocampus, and hypothalamus [92].

Most knowledge about the role of P4 in the CNS derives from in vivo animal studies. Although a strong correlation with humans is assumed, the underlying mechanisms remain incompletely understood. Some studies suggest that P4 has a superior cognitive profile compared to progestins [93].

## 9. Quality of Life (QoL)

The symptoms that have the greatest impact on quality of life for women undergoing menopausal transition are primarily vasomotor, but they also include sleep disturbances, concentration difficulties, and asthenia [94]. These CNS-related symptoms often cluster together [95] and interfere with daily functioning, workplace performance, and domestic wellbeing.

As outlined above, hormone therapy remains the most effective treatment for reducing symptoms and improving quality of life, with benefits observed across most estrogen–progestogen formulations, whether oral or transdermal [96,97]. Women with severe symptoms experience the greatest improvement in quality of life, likely due to the earlier initiation of hormone therapy [98].

Conversely, the WHI study did not find significant health-related quality of life benefits in a subgroup of women aged 50–54 with moderate to severe vasomotor symptoms treated with CEE alone or combined with MPA [99]. However, many scales used in WHI focused on symptom presence rather than on their impact on quality of life. New tools such as the Utian Quality of Life Scale, specifically designed to measure wellbeing distinct from menopausal symptoms, may reveal quality of life improvements even in asymptomatic women [100].

Overall, studies show similar outcomes across HRT formulations regarding vasomotor symptoms and body weight/composition. However, limited evidence suggests that P4 may improve quality of life and sleep more effectively than other progestins [101].

## 10. Conclusions

HRT remains the most effective strategy to address menopausal symptoms and support healthy aging. Among the available regimens, E2/P4 are distinguished by their physiological properties and favorable efficacy–safety balance [12,19]. The combined use of progestins with estrogens is recommended for postmenopausal women with an intact uterus to provide endometrial protection [2]. Although a systematic review reported an increased risk of endometrial cancer with P4-containing HRT, primarily based on observational data, randomized controlled trials demonstrate that P4 is able to prevent endometrial hyperplasia, offering protection comparable to progestins when dosed appropriately [1,21,27].

The recent phase 3 randomized placebo-controlled REPLENISH trial confirmed adequate endometrial protection with P4 combined with E2 for controlling menopausal symptoms [19].

Regarding thromboembolic and cardiovascular outcomes, E2, particularly when administered with a transdermal route, is consistently associated with a lower risk of venous thromboembolism and ischemic events compared with CEE [31,35]. Likewise, P4 appears neutral or even beneficial for coagulation and cardiovascular markers, whereas several progestins demonstrate prothrombotic or atherogenic profiles [32,33,34,35,36,37,38].

Breast cancer remains a primary concern in decision-making counseling for HRT. Current evidence indicates that E2/P4 combinations are associated with a lower risk of breast cancer compared with regimens containing synthetic progestins [47,48,49,50,51,52]. This observation is biologically plausible given the distinct receptor interactions of bioidentical progesterone [53,54].

Considering bone health, HRT containing E2 and P4 has a key role in preventing osteoporosis onset, improving BMD, and reducing fracture risk, with outcomes comparable to those observed with HRT formulations containing synthetic progestins.

Regarding the impact on the CNS, E2 shows neuroprotective properties and reduces the risk of Alzheimer’s disease [78,79,80,81,82,83,84,85,86], while P4 provides additional benefits by improving sleep quality and supporting central nervous system function [87,88,93]. Consistent improvements in quality of life, particularly in women experiencing severe vasomotor and neuropsychological symptoms, have been documented across multiple studies [94,95,96,97,98,99,100,101].

Overall, current evidence supports E2/P4-based HRT as an effective therapeutic option for women with menopausal symptoms, offering an advantageous balance between symptom relief and safety. Nevertheless, most of the current evidence is derived from observational studies, which are inherently susceptible to residual confounding and exposure misclassification. These methodological limitations should be considered when interpreting comparisons between E2/P4 and other regimens, emphasizing the importance of further randomized controlled trials to substantiate existing findings and delineate regimen-specific effects across female subgroups.

## Figures and Tables

**Table 1:** Receptorial effects of P4 and progestins.

Progesterone and Progestins	Antiestrogenic	Estrogenic	Androgenic	Antiandrogenic	Glucocorticoid	Antimineralocorticoid
*Progesterone*	++	-	-	(+)	+	+
*Cyproterone acetate*	+	-	-	+	+	-
*Medroxyprogesterone*	+	-	(+)	-	+	-
*Dydrogesterone*	+	-	-	-	?	(+)
*Norethisterone*	+	+	+	-	-	-
*Levonorgestrel*	+	-	+	-	-	-
*Dienogest*	+	-	-	+	-	-
*Drospirenone*	+	-	-	+	?	+
*Nomegestrol*	+	-	-	+	-	-

++ = strongly effective, + = effective, (+) = weakly effective, - = ineffective, and ? = unknown.

**Table 2:** RR of VTE based on the type of estrogen, on the route of administration, and on the association with different progestogens (modified from reference [31]).

Estrogens, Progestogens, and Their Routes	VTE RR
**Oral preparations**	
Estrogen only	1.42
CEE	1.50
E2	1.31
Combined with progestogen	1.73
CEE combined	1.90
CEE + MPA	2.22
CEE + levonorgestrel	1.59
E2 combined	1.61
E2 + MPA	1.51
E2 + dydrogesterone	1.19
E2 + norethisterone	1.69
E2 + other progestogens	1.40
**Transdermal preparations**	
E2 only	0.94
E2 combined	0.84
